# A new anthropometric model for body composition estimation: Comparison with a bioelectrical impedance consumer device

**DOI:** 10.1371/journal.pone.0271880

**Published:** 2022-09-01

**Authors:** Nicolaus Dahlmann, Vera Demond

**Affiliations:** 1 Institut für Klinische Chemie, Universitätsklinikum Schleswig-Holstein, Kiel, Germany; 2 Endokrinologie, Diabetologie und Stoffwechselmedizin, Universitätsklinik Bonn, Bonn, Germany; University of Mississippi Medical Center, UNITED STATES

## Abstract

**Background:**

The present study refers to an anthropometric model, Dahlmann-Body-Analysis (DBA), based on formerly developed weight-height-frame tables. Including the anthropometric variable abdomen circumference (AC), a further differentiation into muscularity and fat mass could be realized. This enables to calculate the individual percentage fat mass (%FM), providing a cost-effective method for epidemiological studies. The present work sets out to investigate, whether %FM computed by the DBA model compares to BIA measurements, notably under conditions of heavy obesity.

**Subjects/methods:**

In 103 adults (37 males, 66 females, age 42.5 ± 12.5 years, BMI 38.2 ± 4.8 kg/m²) %FM was estimated by a tetrapolar BIA device and compared to results derived by the DBA system. Bland-Altman and simple linear regression analyses were used to determine agreement between methods.

**Results:**

The mean %FM estimates of men (women) ± standard deviation were 36.1 ± 4.8 (49.7 ± 4.5) for BIA and 36.7 ± 4.2 (49.1 ± 4.9) for DBA measurements. Pearson correlation coefficients (r) and Lin’s concordance correlation coefficient (CCC) were r = 0.86 and CCC = 0.84 for men and r = 0.85 and CCC = 0.83 for women, respectively. Bland-Altman plot showed limits of agreement between DBA and BIA %FM that ranged from 5.5% to -4.3% for men and 4.6% to– 5.7% for women, respectively. Correlations between values measured by both methods were high and the observed confidence interval (SD of the difference between DBA and BIA result multiplied by 2) was low. No systematic error was found. The DBA system overestimates FM by 0.63 ± 4.98 (2SD) % for men and underestimates FM by -0.56 ± 5.28 (2SD) % for women, respectively, compared to the BIA results. The result for both genders is -0.066 ± 5.17 (2SD) %FM. Over all, there is a strong concordance and reproducibility between the DBA and BIA data sets.

**Conclusions:**

The implementation of the abdomen circumference (AC) into the DBA model as a proxy for body fat (%FM) resulted in a strong concordance with BIA measurements. These findings indicate that the DBA model may reflect the body shape of severely obese white European patients with regard to body composition.

## Introduction

The human body is mainly composed of four molecular-level components: water, fat, proteins and minerals, usually in that order of decreasing amounts [1]. The substance that has attracted the most attention, from laypeople to medical professionals, is body fat. Excessive body fat is associated with the occurrence of clinical complications that compromise the quality of life and survival of individuals, such as diabetes, hypertension, cardiovascular disease, musculoskeletal disorders, and cancer [2].

The most widely used way to estimate body fat is the body mass index (BMI)—body weight normalized by height squared (kg/m²). Being a very simple and inexpensive method, it is the basis for WHO’s definition of overweight (25 ≤BMI <30) and obesity (BMI ≥30). However, for a given BMI, the percentage of fat mass (%FM) changes with age, and the rate of this change is different depending on sex, ethnicity and individual differences [3]. And while BMI correlates with fat accumulation and metabolic health in large populations, it is insensitive to the actual distribution of body fat of an individual [4] because adipose tissue is, by far, the most varying compartment between individuals, but also within an individual over time.

Other anthropometric measures, such as waist circumference (WC) and waist-to-hip ratio (WHpR), as a tool to identify those at risk of metabolic complications have received attention worldwide for being strongly associated with several chronic diseases [5–7]. WC and WHpR reflect visceral fat, hence abdominal obesity. A special equation deduced from hip circumference is BAI (body adiposity index) as an alternative to BMI [8]. However, the comparison with ADP (air displacement plethysmography) demonstrated a positive bias of 5.1%FM with a range of 21.8%. Accordingly, the authors conclude that BAI does not provide an accurate estimate of %FM for severely obese patients [9].

Clinical assessments of %FM in severely obese patients are challenged by the high cost of available methods and the lack of accuracy that some of these methods provide under the elevated fat, total and extracellular water, and change in electrolyte concentration exhibited by severely obese patients [10].

Aiming to solve this issue, we proposed a new anthropometric model, named Dahlmann-Body-Analysis (DBA). The system is based on measurements taken by W. Schlegel and his assistant G. Hopfeld on 1749 young adults aged 18 to 30 for men and 17 to 30 for women, all living in Hamburg, Germany, and the surrounding area, being a random sample of the population. The measurements of gender, age, height (Ht), weight (W) and hand circumference (HdC) were carried out between 1955 and 1973, at a time when neither junk nor fast food and the corresponding restaurant chains existed in Germany. The hand circumference (HdC) was implemented into the DBA model as a proxy for skeleton frame and gave for the first time the chance to develop weight-height-frame tables [11]. A measure of frame allows the discrimination between those, who are heavy because of large fat free mass from those whose overweight is largely fat. Deduced from this reference population and based on a multiple regression equation including the parameters height, weight and hand circumference the DBA system allows for each individual the calculation of a reference weight (RefW), which should replace the terms “desirable” or “ideal”.

The “Schlegel” material was compared with conscripts of the German Armed Forces and with school girls measured by the Hamburg Department of Health, respectively, indicating that the DBA model may reflect the body shape of the German population at that time [12].

The limitation of the model in its present form is that it cannot distinguish between muscularity and fat tissue. For that reason, the circumference of the abdomen (AC) as a marker for central obesity is integrated into the DBA model. The processed data of the modified DBA model represent the percentage of fat mass (%FM) and are compared with the results of a low budget tetrapolar eight-electrode Bio Impedance Analysis (BIA) device using eight tactile electrodes to give evidence if the DBA model fulfils the precondition of a clinical trial. Subjects suffering of severe obesity were chosen to be from a theoretical point of view the most challenging and from a clinical point of view the most interesting cohort.

The present study sets out to investigate, whether %FM computed by the DBA model compares to BIA measurements, notably under conditions of heavy obesity.

## Materials and methods

### Patients

For this study, data were collected between January 2019 and May 2021 including 103 adult severely obese white European patients (37 males, 66 females), who were candidates for bariatric surgery and each of whom had a BMI ≥30 kg/m^2^, and were recruited from the outpatients´ clinic of the endocrinological department of the University Hospital, Bonn, Germany. Subjects between 18 to 65 years of age were asked to report to the study centre in the morning and 10 h after the last food intake. The exclusion criteria were: cancer patients; clinically detectable oedema; physical amputations and acute diseases of the liver, lung, kidney and heart.

### Ethics statement

All study procedures were performed according to the ethical standards of the World Medical Association’s Declaration of Helsinki and were approved by the medical ethics committee of the Rheinische-Friedrich-Wilhelms-Universität, Bonn, Germany (197/19). Written informed consent was obtained from each patient prior to trial participation.

### Measurements

Body composition was assessed by single-frequency BIA device (Omron BF-511, Kyoto, Japan). The device uses eight electrodes in a tetrapolar arrangement that requires the subject standing on metal footpads in bare feet and grasping a pair of electrodes fixed on a handle with arms extended in front of the chest. The hands touch the electrodes so that the electrode separator is positioned between the middle and ring finger. Manufacturers`equations were used to predict %FM. According to the instruction manual of the manufacturer the accuracy (SEE) is reported by 3.5%.

Body weight was measured to the nearest 0.1 kg using the body weight scale of the BIA device with the patient standing in the centre of the scale platform, bare foot, wearing underwear. During the measuring period batteries were replaced once. Body height (Ht) was obtained with a stadiometer (seca, Hamburg, Germany) with the patient standing, barefoot with the heels together, back upright, and arms stretched next to the body. Hand circumference (cm) was measured by positioning a non-stretchable measuring tape in the horizontal plane over the base joints of the 2nd to 5th finger. The hand should be strained and the thumb splayed. The left hand is chosen for right-handed people, the right hand for left-handed people. The circumference of the abdomen (AC) was measured at the level of the umbilicus with the patients lying supine. Measurements were taken by fitting the tape snugly without compressing the underlying soft tissue. Readings of all measurements were taken to the nearest mm.

The study was performed in a double-blind form. Measurements were taken in Bonn, send to the first author, processed by the Dahlmann-Body-Analysis (DBA) system and send back. Differences of the DBA and the BIA system outside a range of ± 7.5%FM for men and women, respectively, were taken out of consideration, after a second measurement had confirmed the results several months later. Three candidates of each gender are concerned. At the end a data set of 103 subjects remained.

The proper size of the trial was calculated using the formula n = 2(z_α_+z_ß_)²*(s²/d²) based on risk level I (2α = 0,05) and risk level II (ß = 0, 20, meaning a power of 80%). The standard deviation (s) = 5.4% of the BIA device [12] and the difference (d) of measurements is assumed with 5.6% [20], meaning a minimal sample size of 2*15 = 30.

### Statistical methods

Descriptive statistics are presented as means ± standard deviation (SD). Differences between parameters of body composition assessed by different methods were tested by paired samples t-test. Relative agreement between the variables %FM-DBA and %FM-BIA were analysed by linear regression analysis. Pearson`s correlation coefficient (r) was calculated by square root of the determination coefficient R^2^ for relationships between variables. R² is the proportion of the total variance in the dependent variable that is explained by the independent variables. Observed correlation coefficients were compared using a test for equal correlations, which is a ratio that uses Fisher´s z transformation in the numerator and the square root of the sum of the variances in the denominator. Lin’s concordance correlation coefficient (CCC) was used to assess the concordance between the two methods [13]. Analysis according to the Bland-Altman method was created to determine absolute agreement between the %FM assessed by DBA- and BIA-system. The limits of agreement (LOAs), calculated as bias ± SD error hereby express 95% confidence interval of the individual difference [14]. Bias was calculated as result obtained from the difference of the two methods. Confidence interval was calculated as ±2 SD. All statistics were performed in Excel (Office 2019, Microsoft Corporation, USA). Tests not available in Excel were calculated by hand. A p-value < 0.05 was considered statistically significant.

### Data processing

Details of material and formulas were previously described such as the calculation of the reference weight and the classification of the bone structure [11]. The further separation of body weight into muscularity and body fat is based on the measurement of the abdomen circumference (AC). Processed by a couple of algorithms one result is the percentage of fat mass (%FM). The calculation became possible only by the assignment of body fat to the “Schlegel” material. Based on the characteristics of age and body mass index (BMI) for men (24.4 y, 22.4 kg/m²) and women (22.2y, 20.9 kg/m²) the formulas of a couple of authors like Deurenberg [15], Gallagher [3] and Müller [16] could be addressed. The results are summarized in Table 1 with a mean of 15.9%FM for men and a mean of 25.5%FM for women, respectively.

**Table 1:** Assignment of %FM to the "Schlegel" material, derived from different formulas of Caucasian cohorts.

**"Schlegel" Material**		**Men**	**Women**
Age, year		24.4	22.2
BMI, kg/m²		22.4	20.9
**Reference**	**Method**	**%FM**	**%FM**
Gallagher [3]	4c	16.0	25.1
White [4]			
Müller [15]	ADP	15.5	26.5
Caucasian			
Deurenberg [14]	Specific Weight	16.3	24.8
Caucasian			
**Mean (%)**		**15.9**	**25.5**

**Abbreviations:** ADP: Air displacement plethysmography; 4c: 4 Compartment Model; %FM: percentage fat mass; (): formula.

## Results

An entity of severely obese white European persons was analysed with regard to their %FM processed by the DBA system. The results are compared to the %FM data produced on a BIA scale. Basic characteristics and results from different methods of body composition analysis are given in Table 2, stratified by gender.

**Table 2:** Anthropometric and body composition characteristics of obese Europid adults (Mean ±SD).

	Men		Women		All	
	n = 37		n = 66		n = 103	
	Mean	±SD	Mean	±SD	Mean	±SD
**Age**, years	44.1	12.3	41.6	12.6	42.5	12.5
**Height,** cm	179.0	7.7	166.4	6.2	172.6	6.7
**Weight,** kg	115.5	15.3	108.1	16.7	110.8	16.2
**HC**, cm	21.6	1.5	19.4	1.1	20.4	1.3
**AC,** cm	121.9	12.2	118.3	12.1	119.6	12.1
**BMI**, kg/m²	36.0	4.2	38.9	5.1	38.2	4.8
**AC/Ht**, cm/cm	0.68	0.07	0.71	0.07	0.70	0.07
**%FM**, BIA	36.1	4.8	49.7	4.5	44.8	4.6
**%FM**, DBA	36.7	4.2	49.1	4.9	44.7	4.7

A**bbreviations:** BMI, body mass index; AC, abdomen circumference; HdC, hand circumference; Ht, height; %FM, percentage fat mass; BIA, bioimpedance analysis; DBA, Dahlmann-Body-Analysis.

From the entire sample, most of the patients were women (64%), the BMI ranged from 30 to 52 kg/m^2^, with 34% of patients having BMI > 40 kg / m^2^; age ranged from 18 to 65 years. The mean estimates of %FM calculated by DBA- and the BIA-system were 36.7% vs. 36.1% for men and 49.1% vs. 49.7% for women, respectively. The mean values of each sex, tested by paired samples t-test, revealed no statistically significant differences (p > 0.05).

**Fig 1:**
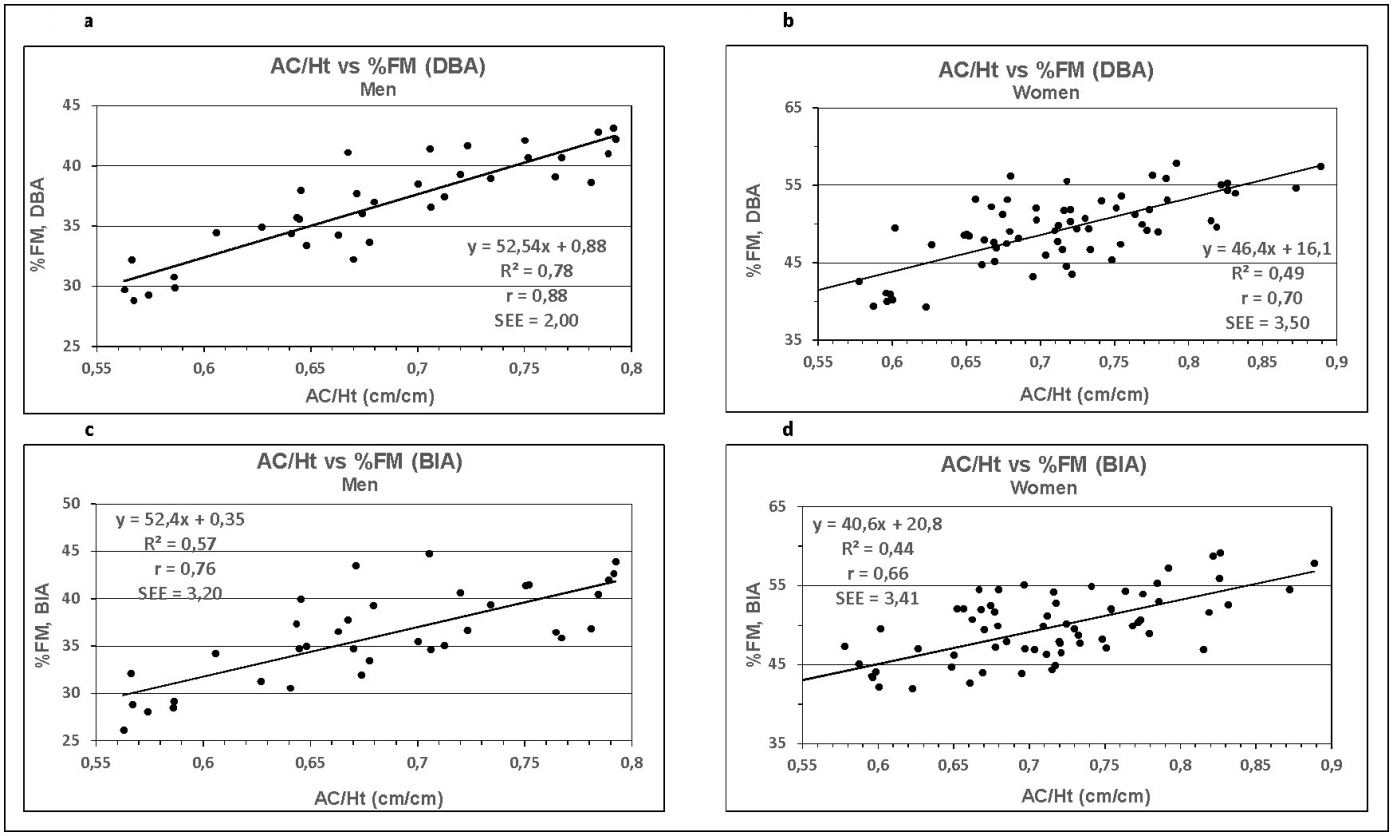
**a-d:** Relationship between AC/Ht vs. %FM derived by DBA and BIA method for men (Fig 1a and 1c) and women (Fig 1b and 1d). Solid line: regression line; R²: determination coefficient; r: Pearson´s correlation coefficient; SEE: standard error of estimate.

Results comparing AC/Ht vs. %FM data, derived according to the DBA system and the BIA measurements, are presented in Fig 1a–1d.

The results are plotted and analysed by regression analysis. The determination coefficient R^2^ and the standard error of estimate (SEE) of the two methods (DBA vs. BIA) are 0.78 (2.00) vs. 0.57 (3.20) for men (Fig 1a and 1c) and 0.49 (3.52) vs. 0.44 (3.41) for women (Fig 1b and 1d). The figures also present Pearson`s correlation coefficients (r), which were calculated for AC/Ht vs. %FM between the two methods and revealed the following results for the DBA system: r = 0.88 for men (Fig 1a) and r = 0.70 for women (Fig 1b). The results obtained from BIA are: r = 0.76 for men (Fig 1c) and r = 0.66 for women (Fig 1d).

**Fig 2:**
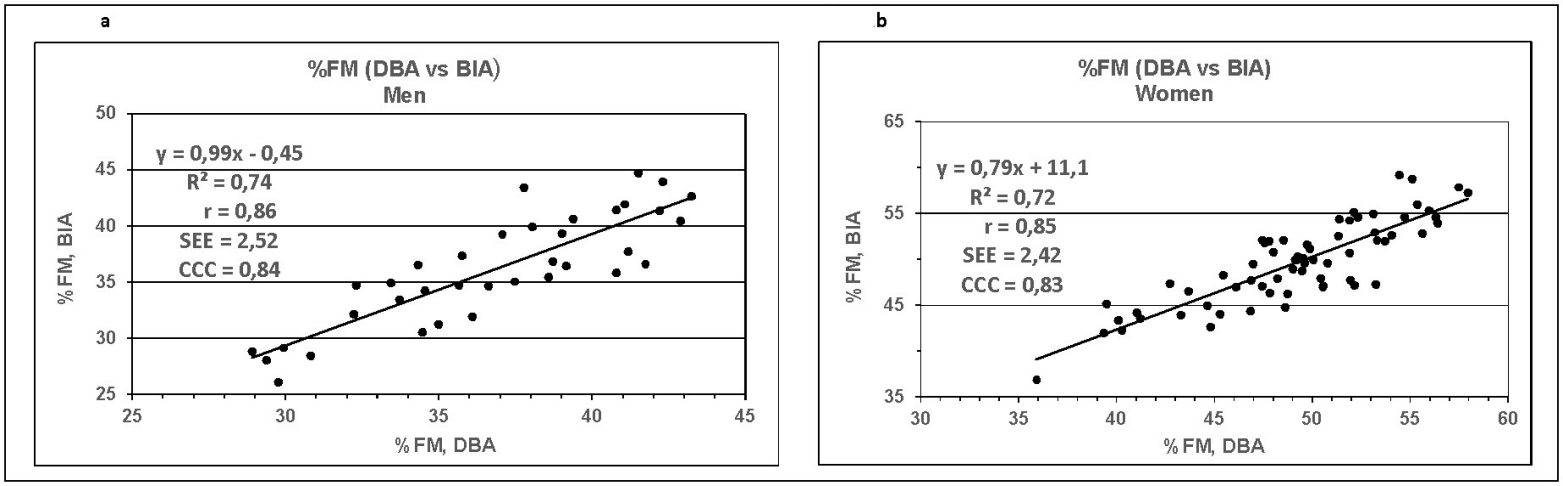
**a and b:** Relationship of %FM derived by BIA and DBA method of men (Fig 2a) and women (Fig 2b). Solid line: regression line; R²: determination coefficient; r: Pearson´s correlation coefficient; SEE: standard error of estimate; CCC: Lin`s concordance correlation coefficients are shown.

The analysis of %FM data assessed by the DBA and BIA method were compared by regression analysis and results are plotted in Fig 2a and 2b.

The determination coefficient R^2^ and the standard error of estimate (SEE) are 0.74 (2.52) for men (Fig 2a) and 0.72 (2.42) for women (Fig 2b). Lin’s concordance correlation coefficients (CCC) revealed the following results: CCC = 0.84 for men and CCC = 0.83 for women, respectively and underline the strong concordance between the two data sets.

**Fig 3:**
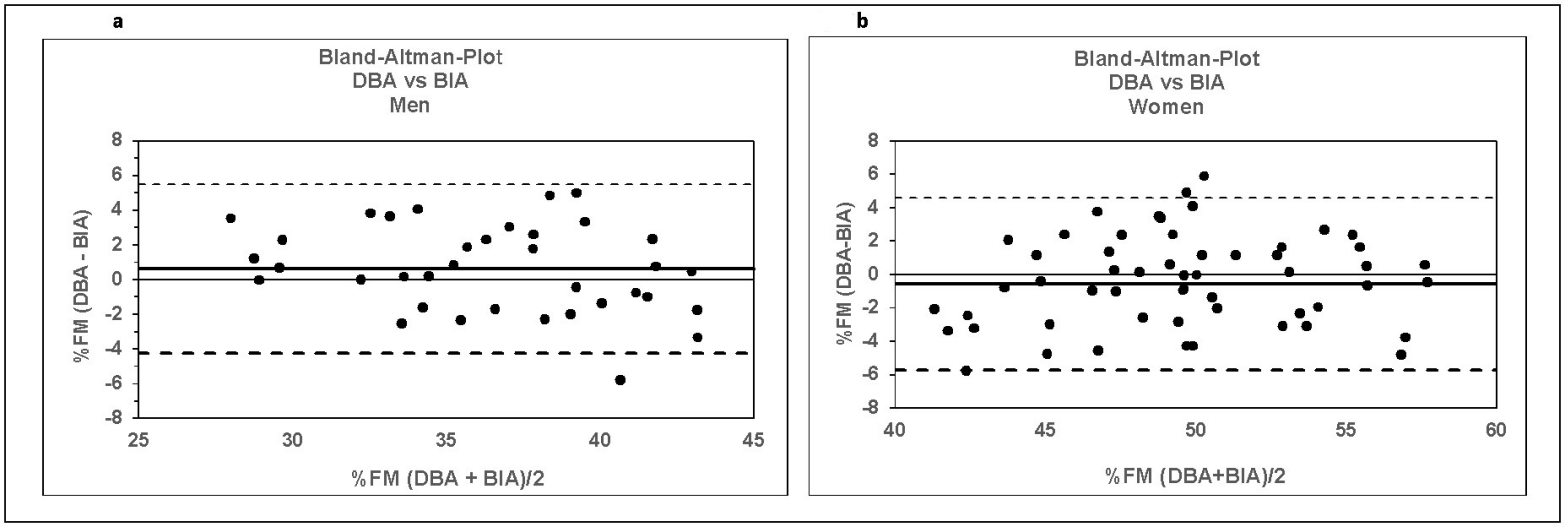
**a and b:** Bland-Altman plot for men (Fig 3a) and women (Fig 3b) showing limits of agreement between the values of %FM estimated by DBA system vs. those derived from BIA measurements. Bold continuous lines indicate the observed average agreement. Continuous lines indicate the line of perfect average agreement. Dashed lines indicate 95% limits of agreement.

Analysis according to the Bland-Altman method was created to determine absolute agreement between the %FM assessed by DBA and BIA system. The limits of agreement between DBA and BIA %FM were 5.5% to -4.3% (range: 9.8%) for men and 4.6% to– 5.7% (range: 10.3%) for women, respectively, as shown in Fig 3a and 3b.

Correlations between values measured by both methods were high and the observed confidence interval (SD of the difference between DBA and BIA result multiplied by 2) was low. No systematic error was found. The DBA system overestimates FM by 0.63 ± 4.98 (2 SD) % for men and underestimates FM by -0.56 ± 5.28 (2 SD) % for women, respectively, compared to the BIA results. The result for both genders is -0.066 ± 5.17 (2SD) %FM. Overall, the goodness of fit and the bias between the two methods are for both genders almost equal.

## Discussion

Usually, anthropometric assessment of body composition relies on prediction equations derived from gold-standard methods. Commonly used prediction equations, including Durnin and Wormsley`s [17] and Jackson and Pollock`s equations [18] have been developed in white European populations. Others were developed to predict anthropometric variables in a sample of Indian adults [19]. This study refers to an anthropometric model, Dahlmann-Body-Analysis (DBA), based on a reference population [11]. Including the anthropometric variable AC, a further differentiation into muscularity and fat mass could be realized. The present study reports on %FM results, using BIA as a reference method.

In this study we used a BIA device that proved to be the best, compared to three other devices like Tanita, Soehnle and Omron BF-400. No bias and narrow limits of agreement were observed for the Omron BF-500 when compared to DXA measures [20]. The instrument is a tetrapolar eight-electrode BIA device using eight tactile electrodes and incorporates proprietary algorithms with no explanation as to their measurement derivation or refence population. That means, the values were obtained from manufacturer derived equations not available to the general public. The bias ±2 SD for %FM of both genders as result obtained from DXA minus BIA (Omron BF-500) measurement is reported as -0.99 ± 5.60 (BMI = 25.8) (20). In comparison to our data (-0.066 ± 5.17; BMI = 38.2) the value of the DBA method is in the same order of magnitude as shown for the DXA measurement.

BIA includes potential error sources like variations in limb length, recent physical activity, nutrition status, tissue temperature and hydration, blood chemistry, ovulation and electrode placement, as reviewed in [21, 22]. Notably the measure of body composition for individuals is regarded to be not informative [23] and especially foot-to-foot devices produce large errors in estimates [20]. Limits of agreement are too wide for accurate application at the individual level, especially in those populations with altered body geometry or fluid compartmentalization [21].

Therefore, abdominal circumference (AC) or waist circumference (WC) should not be overlooked as a valuable tool in assessing central adiposity. In our study, the DBA model reveals high correlation coefficients between %FM and AC/Ht for men (0.88) and women (0.70). This is in line with other studies, which found the waist-to-height ratio to be best predictor of %FM and visceral adipose tissue mass in men and women [24]. As the waist or abdominal circumference is integral part of the DBA algorithms, it offers the chance for further development of visceral fat determination.

However, as pointed out before, there is no universally accepted method of measuring WC [25, 26]. In this study, it was the intention to get the maximal circumference with the measuring tape at the level of umbilicus as a landmark with the subjects lying supine to determine the most appropriate site that best reflects one´s body composition as indicated in percentage body fat in each sex. This assumption is consistent with existing literature that provides evidence that waist-to-height values calculated from AC were significantly greater than the values for WC in both sexes indicating that the umbilical measurement increases the ´fat-sensitive´ nature of the index and should be used for early screening purposes [26, 27].

For epidemiological studies there is a need for a valid body composition measure to be cost-effective, non-invasive, highly reproducible, most convenient and easy to use. It should enable a person for clinical or personal use to evaluate the body weight in terms of health risk. The present study rolls out the means on the basis of sex, height, weight, skeleton frame and body fat.

Study weakness includes possible limited generalizability of the results to populations other than those included in this study. Future research should involve individuals with a wider weight range and other ethnicities.

## Conclusion

The DBA model was shown to be a non-invasive and inexpensive method for calculating %FM. It is based on the abdomen circumference (AC) measurement using the umbilicus as a landmark. The study reported on a bouquet of algorithms to estimate body composition, here %FM, in severely obese patients with a validity and precision that matches that of an eight-electrode BIA device. DBA exhibited a good performance in obese patients with a BMI greater than 30 kg/m². Future research is recommended to confirm the present findings across different ethnic and weight groups.

## Supporting information

S1 Data DBA-BIA, men. (XLSX)

S2 Data DBA-BIA, women. (XLSX)

## Abbreviations

BMI: body mass index
CCC: Lin’s concordance correlation coefficient
DBA: Dahlmann-Body-Analysis
Hd: hand
Hp: hip
Ht: height
r: Pearson correlation coefficient
W: weight
